# Damnacanthal is a potent inducer of apoptosis with anticancer activity by stimulating p53 and p21 genes in MCF-7 breast cancer cells

**DOI:** 10.3892/ol.2014.1898

**Published:** 2014-02-20

**Authors:** MUHAMMAD YUSRAN ABDUL AZIZ, ABDUL RAHMAN OMAR, TAMILSELVAN SUBRAMANI, SWEE KEONG YEAP, WAN YONG HO, NOR HADIANI ISMAIL, SYAHIDA AHMAD, NOORJAHAN BANU ALITHEEN

**Affiliations:** 1 Department of Cell and Molecular Biology, Faculty of Biotechnology and Biomolecular Sciences, Universiti Putra Malaysia, Serdang, Selangor 43400, Malaysia; 2 Institute of Bioscience, Universiti Putra Malaysia, Serdang, Selangor 43400, Malaysia; 3 Faculty of Applied Sciences, Universiti Teknologi Mara, Shah Alam, Selangor 40450, Malaysia; 4 Department of Biochemistry, Faculty of Biotechnology and Biomolecular Sciences, Universiti Putra Malaysia, Serdang, Selangor 43400, Malaysia

**Keywords:** damnacanthal, MCF-7, anticancer, caspase, p53, p21, apoptosis

## Abstract

Damnacanthal, an anthraquinone compound, is isolated from the roots of *Morinda citrifolia L.* (noni), which has been used for traditional therapy in several chronic diseases, including cancer. Although noni has long been consumed in Asian and Polynesian countries, the molecular mechanisms by which it exerts several benefits are starting to emerge. In the present study, the effect of damnacanthal on MCF-7 cell growth regulation was investigated. Treatment of MCF-7 cells with damnacanthal for 72 h indicated an antiproliferative activity. The MTT method confirmed that damnacanthal inhibited the growth of MCF-7 cells at the concentration of 8.2 μg/ml for 72 h. In addition, the drug was found to induce cell cycle arrest at the G1 checkpoint in MCF-7 cells by cell cycle analysis. Damnacanthal induced apoptosis, determined by Annexin V-fluorescein isothiocyanate/propidium iodide (PI) dual-labeling, acridine-orange/PI dyeing and caspase-7 expression. Furthermore, damnacanthal-mediated apoptosis involves the sustained activation of p21, leading to the transcription of p53 and the Bax gene. Overall, the present study provided significant evidence demonstrating that p53-mediated damnacanthal induced apoptosis through the activation of p21 and caspase-7.

## Introduction

Breast cancer is the most common type of malignant tumor and the second highest mortality among females with cancer (1). Survival rates for breast cancer have greatly increased with significant improvement in surgical technology and therapy regimens over the last three decades, particularly for early-stage breast cancer. However, no effective treatments currently exist for metastatic breast cancer (2,3). As cancer development largely results from the uncontrolled growth of malignant cells, in which cell proliferation surpasses cell death, deregulation of apoptosis, which occurs frequently in a vast majority of cancer types, has become a non-negligible target for anticancer strategies (4,5). Proapoptotic compounds, derived from synthetic chemistry or natural sources, are also under active investigation for their therapeutic effects and for their mode of actions against various types of cancer. Over the past decades, considerable effort has been directed to using natural products as a source of novel anticancer drugs in the fight against the challenge that a number of types of cancer remain incurable by currently available therapeutic approaches.

*Morinda citrifolia L.* (Rubiaceae), commonly known as noni, is a small evergreen tree or shrub that is widely distributed throughout the pacific islands, Southeast Asia and other tropical and semitropical areas. It has been widely used in therapeutic preparations for centuries, owing to its anti-inflammatory, antibacterial, antiviral, antifungal and antitumor properties (6–9). Damnacanthal, an anthraquinone compound, was isolated from the roots of *Morinda citrifolia L*. and identified as a potent inhibitor of different types of human tumor cells, including human T-lymphoblastic and acute promyelocytic leukemia (10) and breast carcinoma (11). These anticancer effects of damnacanthal were identified through interfering with the cell cycle, inducing apoptosis and inhibiting the invasive potential of cancer (12–14). However, the underlying molecular mechanisms of these anticancer effects remain under investigation. In the present study, the human breast cancer MCF-7 cell line was used to investigate the effects of damnacanthal on the breast cancer cell proliferation, apoptosis, cell cycle arrest and apoptotic gene expression.

## Materials and methods

### Compound

**Figure 1:**
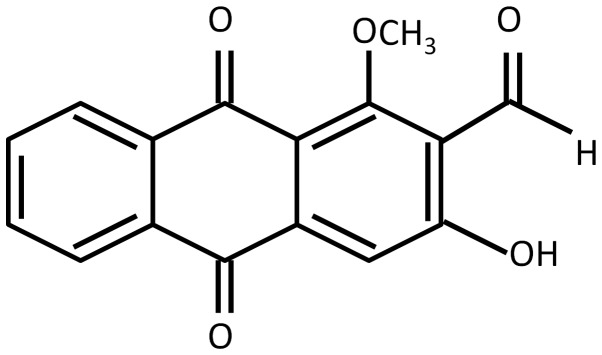
Chemical structure of damnacanthal.

Damnacanthal was obtained from the Faculty of Applied Sciences, Mara University of Technology (Shah Alam, Malaysia) and used as received. The structure of damnacanthal is shown in Fig. 1.

### Cell culture

MCF-7 cells, a human breast cancer cell line obtained from the American Type Culture Collection (Rockville, MD, USA), were cultured as monolayers in RPMI-1640 medium (Sigma-Aldrich, St. Louis, MO, USA) containing 10% (vol/vol) fetal bovine serum (Gibco, Grand Island, NY, USA), 100 U/ml penicillin (Sigma-Aldrich) and 100 μg/ml streptomycin (Sigma-Aldrich), at 37°C in a humidified environment containing 5% CO_2_ and 95% air.

### Cytotoxicity assay

The MCF-7 cells were seeded in 96-well microtiter plates (5,000 cells/well). Following 24 h of incubation in the appropriate medium, cells were treated with various concentrations of damnacanthal for an additional 72 h of culture. Next, 20 μl stock MTT solution (Calbiochem, Darmstadt, Germany) was added to each well (final concentration, 0.5 mg/ml) for an additional 4 h of incubation (37°C; 5% CO_2_). Then, 200 μl dimethylsulfoxide (Sigma-Aldrich)was added to each well and the absorbance at 570 nm was determined. By MTT method, cell numbers were obtained as absorbance values at 570 nm. The results were expressed as viability compared with that of vehicle-treated cells. Each treatment had three independent plates and the representative results were reproducible in three independent experiments. The selectivity of the cytotoxicity of damnacanthal on the MCF-7 cell line was also evaluated.

### Quantification of apoptosis

Damnacanthal-induced cell death in MCF-7 cells was quantified using propidium iodide (PI) (Sigma-Aldrich) and acridine-orange (AO, Sigma-Aldrich) double staining according to standard procedures and examined under fluorescence microscope (Eclipse Ti, Nikon, Melville, NY, USA). Briefly, treatment was performed in a 25-ml culture flask. MCF-7 cells were plated at a concentration of 1×10^6^ cells/ml and treated with damnacanthal at IC_50_ concentration. Flasks were incubated in an atmosphere of 5% CO_2_ at 37°C for 72 h. The cells were then spun down at 1,000 × g for 10 min. Supernatant was discarded and the cells were washed twice using phosphate-buffered saline (PBS) following centrifugation at 1,000 × g for 10 min to remove the remaining media. In total, 10 μl fluorescent dyes, AO (10 μg/ml) and PI (10 μg/ml), were added into the cellular pellet at equal volumes. Freshly stained cell suspension was dropped onto a glass slide and covered by a coverslip. Slides were observed under ultraviolet (UV)-fluorescence microscope within 30 min prior to the fading of the fluorescence color. All the treatments and time points were performed in three individual experiments. AO and PI are intercalating nucleic acid-specific fluorochromes that emit green and orange fluorescence, respectively, when bound to DNA. Of the two, only AO crosses the plasma membrane of viable and early apoptotic cells. Viewed by fluorescence microscopy, viable cells appeared to exhibit green nuclei with intact structures, while apoptotic cells exhibited bright-green nuclei showing condensation of the chromatin as dense green areas. Late apoptotic cells and necrotic cells were stained with AO and PI. Comparatively, PI produces the highest intensity emission. Thus, late apoptotic cells exhibited orange nuclei showing condensation of the chromatin, whilst necrotic cells exhibited orange nuclei with intact structures.

### Annexin V and PI staining for cell death analysis

For assaying the influence of damnacanthal on the induction of cellular apoptosis and cell death, MCF-7 cells in exponential growth were treated with IC_50_ concentrations of damnacanthal for 72 h. Assessment of apoptotic and dead cells was performed by Annexin V and PI staining. Following incubation, cells were washed twice with PBS and the cell pellet was resuspended with Annexin V binding buffer followed by incubation with Annexin V conjugated to fluorescein isothiocyanate (FITC) and PI staining solution (BD Pharmingen, San Diego, CA, USA) for 5 min at room temperature in the dark. Cells were then analyzed by flow cytometry within 1 h. Flow cytometry was performed on FACScan (BD Biosciences, San Jose, CA, USA).

### Cell cycle analysis

For cell cycle analysis, 5 ml MCF-7 cells (5×10^4^ cells/ml) were cultured in 25-cm^2^ flasks, with or without damnacanthal at IC_50_ concentration for 72 h. Cells were then harvested, washed in PBS, centrifuged and resuspended in 1 ml sodium citrate (0.1%) containing 0.05 mg PI and 50 μg RNase for 30 min at room temperature in the dark. Flow cytometry was performed on FACScan (BD Biosciences), with collection and analysis of the results performed using CellQuest software (BD Biosciences).

### Total RNA preparation and reverse transcription-polymerase chain reaction (RT-PCR) analysis

The apoptosis-related genes were analyzed following the treatment of the MCF-7 cells with or without damnacanthal at IC_50_ concentration for 72 h. The cells were trypsinized and washed twice with PBS. Total RNA was prepared using a Qiagen RNA extraction kit (Qiagen, Hilden, Germany). The RNA concentration was determined by reading the absorbance at 260 and 280 nm with a UV spectrophotometer (DU730; Beckman Coulter, Petaling Jaya, Selangor, Malaysia). The total RNA was transcribed to cDNA using the GenomeLab™ GeXP start kit (Beckman Coulter, Miami, FL, USA) for RT-PCR, according to the manufacturer’s instructions. The following primers were designed from the known sequences: 5′-CCCTTTTGCTTCAGGGTTTC-3′ and 5′-ACAAAGTAGAAAAGGGCGACAA-3′ for Bax; 5′-TGTGGACCTGTCACTGTCTTG-3′ and 5′-TAGGGCTTCCTCTTGGAGAA-3′ for p21Cip1; 5′-CAGACCGGTCCTCGTTTGTA-3′ and 5′-ACCTCG GCATCTTTGTCTGTT-3′ for caspase-7; and 5′-AAGGTG AAGGTCGGAGTCAA-3′ and 5′-AGATCTCGCTCCTGG AAGATG-3′ for GAPDH. The amplification profile was as follows: Denaturing at 94°C for 30 sec; annealing at 55°C for 30 sec; and extension at 68°C for 1 min. The cDNA was amplified using MJ Research PTC-225 analyzer (MJ Research Inc., St. Bruno, QC, Canada) for 35 cycles, followed by a step of 10 min at 72°C to extend the partially amplified products. These cycling conditions were established empirically to provide a linear increase in product intensity proportional to the amount of template. The PCR products with fluorescently labeled fragments were separated by capillary gel electrophoresis (One Capillary array, BD Biosciences, Franklin Lake, NJ, USA), at 6.0 kV and 50°C for 35 min, according to their product size, and the results were analyzed using GenomeLab GeXP system software (Beckman Coulter). GADPH was selected as the reference gene for normalizing all results of the targeted genes.

### Analysis of Bcl2, p53, estrogen receptor (ER)-α and X-linked inhibitor of apoptosis protein (XIAP) expression by flow cytometry

Cells were seeded at a density of 5×10^5^ cells/well in six-well culture plates. Following treatment with IC_50_ concentrations of damnacanthal for 72 h, cells were harvested and fixed by BD Cytofix/Cytoperm™ Fixation/Permeabilization (BD Biosciences). The cells were stained with fluorochrome-conjugated monoclonal anti-mouse p53 and Bcl-2 (Santa Cruz Biotechnology, Inc., Santa Cruz, CA, USA), XIAP (BD Biosciences) and ER-α (Abcam, Cambridge, MA, USA). The cells were washed twice with PBS to remove non-specific binding stained with secondary fluorochrome-conjugated monoclonal antibody mouse anti-p53, -Bcl2, -XIAP and -ER-α (Abcam). The cells were washed twice and analyzed by BD FACSCalibur multicolor flow cytometer (BD Biosciences).

### Statistical analysis

Data are presented as the mean ± standard deviation. P-values were determined by analysis of variance followed by Student-Newman-Keuls test for multiple comparisons. P<0.05 was considered to indicate a statistically significant difference.

## Results

### Effect of damnacanthal on the viability of MCF-7 cells

**Figure 2:**
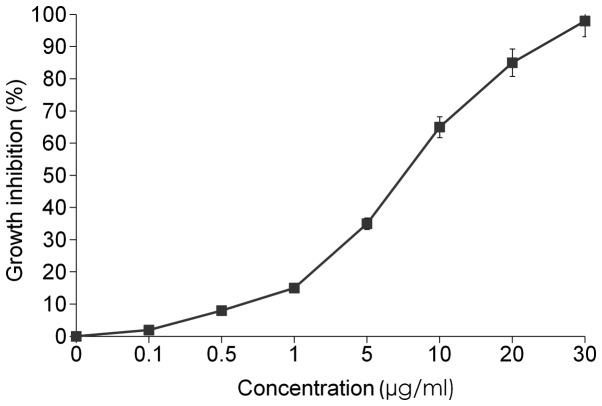
Effect of damnacanthal on growth of breast cancer MCF-7 cell line. Cells were seeded in 96-well plates and incubated with various concentrations of damnacanthal for 72 h at 37°C. Cell viabilities were determined by MTT assay. Data points are presented as the means ± standard deviation of triplicate experiments.

To examine the effects of damnacanthal on MCF-7 cell viability, MTT assay was performed. MCF-7 cells were plated onto 96-well plates and treated with various concentrations of damnacanthal (0–30 μg/ml) for 72 h. As shown in Fig. 2, damnacanthal dose-dependently (P<0.05) inhibited cell viability. These inhibitory effects were observed following incubation with 8.2 μg/ml damnacanthal, reducing cell growth by 50% (IC_50_).

### Damnacanthal induced apoptotic cell death in MCF-7

**Figure 3:**
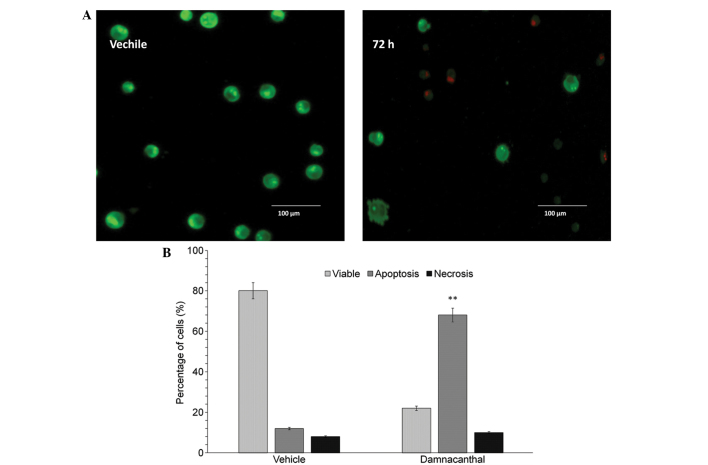
(A) Morphological assessment of MCF-7 cells stained with acridine orange (green) and propidium iodide (red). Cells were incubated with or without damnacanthal at IC_50_ concentration for 72 h. Cells with intact membranes and stained green indicated viable cells; cells that showed chromatin condensation, nuclear genome fragmentation and membrane blebbing indicated early apoptosis; and cells that were stained orange and contained fragmented DNA represented secondary necrotic or late apoptotic cells (magnification, ×100; scale bar, 100 μm). (B) Population of viable, apoptotic and necrotic cells in vehicle-treated MCF-7 cells (control) and MCF-7 cells treated with damnacanthal for 72 h. ^**^P<0.05 vs. vehicle-treated group.

AO and PI dyes were used to differentiate viable, apoptotic and necrotic cells under fluorescence microscope. Fig. 3A shows the intact viable cells and apoptotic and necrotic cells following the treatment of MCF-7 cells with damnacanthal at IC_50_ concentration for 72 h. The apoptotic event of damnacanthal-treated cells was increased significantly: ≤4-fold higher than the vehicle-treated cells (Fig. 3B). A fraction of necrotic cells were also detected in the treatment group.

### Induction of apoptosis by damnacanthal in MCF-7 cells

**Figure 4:**
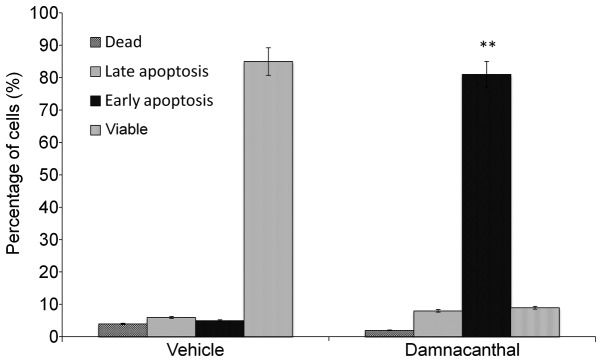
Flow cytometry analysis of MCF-7 cells, vehicle-treated (control) or treated with damnacanthal for 72 h, stained with Annexin V-fluorescein isothiocyanate/propidium iodide. Data are presented as the means ± standard error of the mean for three assays, each in triplicate. ^**^P≤0.05, vs. vehicle-treated group, determined by analysis of variance.

To determine whether the loss of cell viability induced by damnacanthal was associated with apoptosis, Annexin V-FITC/PI binding assay was performed. The assay evaluates phosphatidylserine turnover from the inner to the outer lipid layer of the plasma membrane, an event typically associated with apoptosis. Flow cytometric analysis revealed that the percentage of apoptotic cells with Annexin V-positive but PI-negative cells increased gradually with concentration in damnacanthal-treated cells. As shown in Fig. 4, following 72 h of treatment with damnacanthal at IC_50_ concentrations, the numbers of apoptotic MCF-7 cells, as revealed by Annexin V binding, increased in a dose-dependent manner, indicating a proapoptotic activity of damnacanthal. The proportion of MCF-7 cells in early apoptosis was 80.6% after 72 h (P<0.05, vs. vehicle-treated cells at the same time). For cells that were in the late apoptosis, the proportion was 8.1% at 72 h, while the proportion of the cells in necrosis was 2% at 72 h (P<0.05, vs. vehicle-treated cells at the same time). For the vehicle-treated MCF-7 cells, the percentage of cells that underwent necrosis was <4% at 72 h incubation time (Fig. 4).

### Damnacanthal induces G1 cell cycle arrest

**Figure 5:**
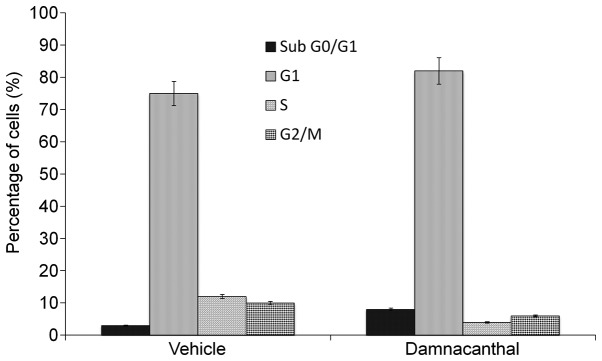
Flow cytometry analysis of MCF-7 cells treated with and without damnacanthal for 72 h stained with propidium iodide. DNA contents were analyzed by flow cytometry and apoptosis was measured by the accumulation of sub-G1 DNA contents in cells and compared with vehicle-treated MCF-7 cells. Results are representative of three independent experiments. Data are presented as the means ± standard deviation of the percentage of cells in individual phases of the cell cycle from four independent experiments.

Following the treatment of MCF-7 cells with 8.2 μg/ml damnacanthal for 72 h, the cell population in G1 phase increased to 80% which was accompanied with a decrease in the S (5%) and G2 (8%) phases (Fig. 5). These results clearly indicated that damnacanthal induces post G1-arrest and apoptosis among the treated cells.

### Activation of apoptotic genes by damnacanthal

**Figure 6:**
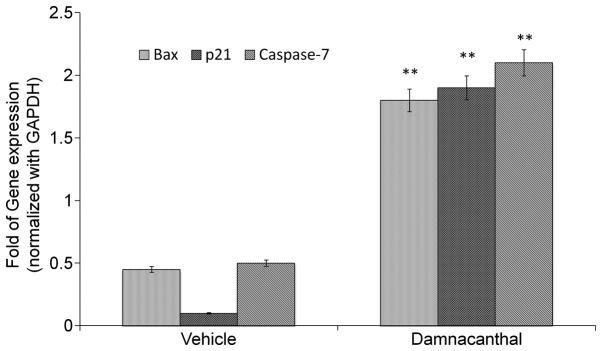
Fold-change of gene expression in MCF-7 cells treated with and without damnacanthal for 72 h were analyzed by reverse transcription polymerase chain reaction. The control cells were treated with vehicle in RPMI medium. Results are representative of three independent experiments. Data are presented as the means ± standard deviation of four independent experiments. ^**^P<0.05 vs. vehicle-treated group.

The expression of proapoptotic genes was analyzed in MCF-7 cells treated with or without damnacanthal for 72 h. The damnacanthal-treated cells showed significant changes in the expression levels of BAX, p21 and caspase-7 genes (Fig. 6). For the p21 gene, all treatments exhibited a significant increase (P<0.05) compared with the control.

### Involvement of apoptotic proteins in damnacanthal-induced apoptosis

**Figure 7:**
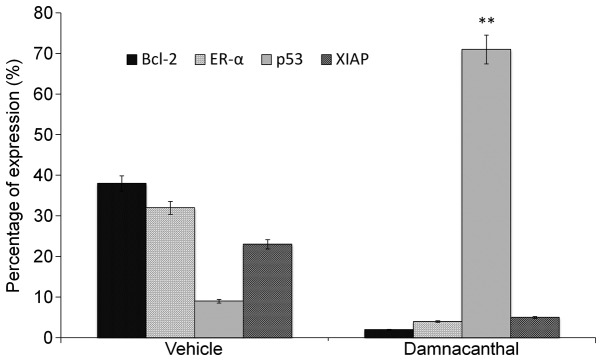
Flow cytometry analysis of MCF-7 cells treated with and without damnacanthal for 72 h were analyzed for the expression of Bcl-2, estrogen receptor-α, p53 and X-linked inhibitor of apoptosis proteins. The control cells were treated with vehicle in RPMI medium. Results are representative of three independent experiments. Data are presented as the means ± standard deviation of the percentage of protein expression from four independent experiments. ^**^P<0.05 vs. vehicle-treated group.

Expression levels of proteins that are involved in pro- and anti-apoptosis, such as Bcl-2, p53, ER-α and XIAP, were evaluated using a multicolor flow cytometer. Fig. 7 shows the percentage changes on the expression level of protein following the treatment of MCF-7 cells with damnacanthal at 8.2 μg/ml for 72 h. The percentage expression level of p53 protein increased significantly (P<0.05) in damnacanthal-treated MCF-7 cells compared with the control. Simultaneously, percentage expression levels of Bcl-2, XIAP and ER-α protein were suppressed significantly (P<0.05).

## Discussion

Cancer has become an increasing public health issue for its high rates of morbidity and mortality. In the current study, the anticancer activity of damnacanthal, an anthraquinone which is extracted from the noni plant, was investigated. Considering that little is known concerning the anticancer activities and related mechanisms of damnacanthal, the current study performed further investigations to elucidate the antitumor activities of damnacanthal in the human breast cancer MCF-7 cell line and the possible mechanisms involved. The experimental results showed that damnacanthal exhibits potent cytotoxicity to MCF-7 cells, with an IC_50_ of 8.2 μg/ml (Fig. 2). Apoptosis assay showed that apoptotic cells induced by damnacanthal exhibited cellular alterations, determined by counting chromatin condensation and nuclear fragmentation by AO/PI (Fig. 3A and C). Annexin V/PI double-staining assay further confirmed the results of the AO/PI staining by showing the important membrane alterations associated with apoptosis in MCF-7 cells and the percentage of apoptosis increase in damnacanthal treatment (Fig. 4). The cell cycle results demonstrated that damnacanthal induced G1 arrest and apoptosis among the damnacanthal-treated cells (Fig. 5). All these results showed that damnacanthal increases antitumorigenic activity by increasing the expression of p53, followed by p21. It has been previously reported that damnacanthal induces apoptosis in a number of cells, including HL-60, MOLT-4, CEM-SS, HT-29, Hela, 3T3 and peripheral blood mononucleated cells (10,15,16). Proposed mechanisms for the proapoptotic effect include activation of caspases, induction of cytochrome *c* release, regulation of protein kinase C isoform expression, inhibition of NF-κB and suppression of activator protein 1 (17–19). The results of the current study were consistent with our previous study, as damnacanthal induced apoptosis in HL-60 and Wehi-3B cells (10). Further investigations were performed to highlight the apoptotic pathways involved in the apoptosis induced by damnacanthal in MCF-7 cells.

Previous studies have revealed that caspases are critical in executing apoptosis (20). In order to gain further insight into the mechanism of the signaling cascade, the present study examined the molecular sequence of events in damnacanthal-induced apoptosis. Apoptosis may occur via two fundamental pathways: i) death receptor or extrinsic pathway; and ii) mitochondrial or intrinsic pathway. The present study demonstrated the considerable role of the mitochondrial apoptotic pathways in apoptosis induced by damnacanthal in MCF-7 cells. Damnacanthal-mediated activation of Bax, p21 and caspase-7 was identified in MCF-7 cells. The activation of p21 and caspase genes stimulates p53 phosphorylation (21). Although multiple pathways contribute to the modulation of p53 (22), the current study investigated the expression of p21 as one of the upstream molecules of p53. The results demonstrated that p21-p53 signaling is one of the key pathways in mediating damnacanthal-induced apoptosis. In addition, the role of p21 in the transcription of the p53-regulated Bax gene is likely to involve p53 phosphorylation (23). The increased damnacanthal-dependent p53 protein levels are consistent with the damnacanthal-dependent transcriptional induction of Bax. Extensive analyses of damnacanthal-dependent modifications of p53 are in progress to link p21 activity with p53 function in damnacanthal-mediated apoptosis. Although modulation of p21 and p53 signaling is common, the current study established connections between well-known proapoptotic molecules in the damnacanthal-induced apoptosis.

In conclusion, damnacanthal, a bioactive compound from noni roots, enhanced the expression of p21 and caspase-7. Overexpression of p21 directly activates transcription and expression of p53 and, subsequently, increases apoptosis in human breast cancer MCF-7 cells. These results are likely to highlight the potential benefits of damnacanthal for further preclinical or clinical practice and damnacanthal may be a useful cancer prevention/therapeutic agent in human breast carcinoma.
